# Injection Site Tumours and Preceding Pathological Changes in rats Treated Subcutaneously with Surfactants and Carcinogens

**DOI:** 10.1038/bjc.1973.28

**Published:** 1973-03

**Authors:** J. Hooson, P. Grasso, S. D. Gangolli

## Abstract

**Images:**


					
Br. J. Cancer (1 973) 27, 230.

INJECTION SITE TUMOURS AND PRECEDING PATHOLOGICAL

CHANGES IN RATS TREATED SUBCUTANEOUSLY WITH

SURFACTANTS AND CARCINOGENS

.J. HOOSON, P. GRASSO AND S. D. GANGOLLI

From, the British Industrial Biological Research, Association, IVoodnanste i-e Road, Carshaltun,

Surrey

Received 18 October 1972.  Accepted 18 December 1972

Summary.-The sequential histological changes and neoplastic response occurring
in the subcutaneous tissue of rats after injection of surfactants or carcinogens were
compared. Twice weekly subcutaneous injections of the surfactants Blue VRS and
Light Green SF elicited a deranged connective tissue repair with continued prolifer-
ation of fibroblasts and extensive collagen desposition. In contrast, the carcinogens
N-methyl-N-nitrosourea (MNU) and N-nitroquinoline-N-oxide (NQO) appeared
to inhibit connective tissue repair and produce morphologically abnormal fibroblasts.
The spectrum of neoplastic response was also found to differ. Surfactants gave rise
to local sarcomata only after about 47 weeks, whereas carcinogens produced local
sarcomata and adenocarcinomata after 20 and 12 weeks respectively.

WE have previously reported the
results of investigations of the chemical,
physical and biological factors involved
in the production of local sarcomata in
rodents by repeated subcutaneous injec-
tion of a variety of chemicals. The relev-
ance of these sarcomata in terms of the
potential carcinogenic risk of such
chemicals to man have been evaluated
(Grasso et al., 1971; Hooson and CGrasso,
1971). These investigations were based
on a sequential study of the local tissue
reactions to repeated injections and the
analysis of the physical properties of
compounds involved. A correlation was
found between the early lesion induced
in the subcutaneous tissues by a short-
term series of 10-20 injections given at
the same site and the production of local
connective tissue tumouirs by a long-term
series of injections. Histologically, it was
found possible to distinguish 4 types of
reaction (types I IV). Type I and II
reactions were mild and self-limiting and
did not progress to neoplasia. In contrast,
type III and IV reactions were progressive
and proliferative and injection site sar-

comata were invariably produced subse-
quently (Grasso and Golberg, 1966a). It
was found that compounds eliciting a
type III or IV reaction possessed certain
physicochemical properties (surface acti-
vity, hypertonicity, etc.) capable of pro-
(lucing cell injury. Thus it appears that
sarcomata which followed the induction
of a tvpe III or IV reaction were an
indirect result of repeated injury to local
fibroblasts and not due to a process of
direct chemical carcinogenesis.

In contrast, a short-term study of the
lesions induced by repeated subcutaneous
injection of water soluble carcinogens,
whose oncogenic potencies have been
demonstrated by other methods of admin-
istration, gave different results. The
lesion was characterized in all cases by an
inhibition of the normal reparative response
and the appearance of cytologically abnor-
mal cells (Hooson and Grasso, 1971). WAe
felt that it was important at this stage to
extend these observations from the initial
5 weeks and to compare (1) the changes
occurring from this time up to the appear-
ance of tumours (the intermediate period)

INJECTION SITE TUMOURS AND PRECEDING PATHOLOGICAL CHANGES

and (2) the spectrum of neoplastic response
produced by carcinogens with that pro-
duced by non-carcinogenic but irritant
compounds.

Accordingly, 2 surfactant food colour-
ings, Light Green SF and Blue VrRS,
which gave type III and IV responses
respectively on slhort-term test, were
injected in aqueous solutions into rats
twice weekly and the progress of the
lesion examined at frequent intervals.
Two water soluble carcinogens, N-methyl-
N-nitrosourea (MNU) and N-nitroquino-
line-N-oxide (NQO) were administered
under the same experimental regimen
and the tissue reaction during the inter-
mediate period, and the overall neoplastic
response, were compared. These obser-
vations were made over periods of time
up to 60 weeks.

MATERIALS AND METHODS

Anintals.-Details of animals used are
given in Table I, together with the experi-
mental regimens employed. On average rats
weighed 100 g at the start of experiments.
The animals were maintained at a tempera-
ture of 22? ? 1?C and at 500/ relative
humidity: they had free access to Spillers
Small Laboratory animal diet and water.
All animals were inspected twvice weekly and
the injection  site shaved  with  electric
clippers.

Chemicals-Light Green SF sodium salt
and Blue VRS sodium salt w ere obtained
from the Food Colours Committee of the
Chemical Industries Association. The specifi-
cations of these colourings w% ere given in
previous publications (Grasso and Golberg,
1966b). MNU was obtained from K and K
Laboratories Incorporated, Plainview, N.Y.
NQO was supplied by the Daichi Pure
Chemical Co. Ltd., Tokyo, Japan.

Soit?ions. Solutions of all chemicals
were prepared using CO2 free distilled water.
Colourings were filtered and buffered to
pH 7 0 before injection. Aqueous solutions
of NQO and MNU at the concentrations used
in these experiments had a pH of 7-2 and
6-7 respectively. All solutions wNere freshly
prepared before injection.

Details of concentrations, volumes and
frequency of injection are given in Table 1.

All injections were given as far as possible
into exactly the same site, the right flank.

Conduct of experiments. The numbers of
animals used and details of treatment are
shown in Table I. Equal numbers of male
and female animals were used in each experi-
ment. For the sequential study of the
changes in the subcutaneous tissue, 2 animals
were killed 24 hours after the first and every
4th injection up to the termination of Experi-
ments 1 and 2.

In Experiments 3 and 5, groups of 2 rats
were killecl at intervals of 4 injections up to
the 20th and subsequently after every 8th
injection. The subcutaneous tissue at the
injection site was removed, fixed and pre-
pared for histological and ultrastructural
examination as described previously (Hooson
and Grasso, 1971). At the same time inter-
vals, 2 animals in each group received no
further injections in order to observe the
long-term effects of different numbers of
injections. These animals were killed at the
termination of the experiment or earlier if
tumours developed. The injection site was
removed, fixed and prepared for histological
examination as previously mentioned. Ten
male and 10 female rats were injected
subcutaneously twice weekly with 050%
MNU and 01% NQO for a maximum of 35
wAeeks in order to observe the neoplastic
l-esponse (Experiments 4 and 6).

In all cases, animals with tumours were
killed w hen the animals deteriorated in
health or ulceration of the tumour threatened.
WIhen possible, sarcomata were transplanted
into the subcutaneous tissue of 4-6 recipient
animals which were killed 6 weeks later. No
attempt to transplant adenocarcinomata was
made.

RESULTS

Tissue reactions to Light Green SF and
Blue VRS

(a) Rats injected throuyhout the experi-
mnental period. The local lesions produced
by repeated injections of these colourings
(1- 15) have been reported in an earlier
publication (Grasso and Golberg, 1 966a).

The principal features of these lesions
consisted of destruction of the normal
architecture of the rat subcutaneous
tissue and its replacement by scar tissue.
This scar tissue was made up of thick

231

J. HOOSON, P. GRASSO AND S. D. GANGOLLI

"0

FT )

Cl

0

0 -4

0

X

j'QiO

4Q1 O'

* * *

C      I 00   I   I

1-

-0 m 00 C4 "t C)

COeq -    lC0  -q '

N CC 00 0 0

CO 01 00 0 t0 N

1-        -

tsmo Xo o -o

0"-0 000

cca   :   ~   ~ <

p o4

C)  ~ ~ ~ 0

?.o Z   "d
X- ~ z O   Im C

S

0
C)

re)

232

E

00E

z j G
o     lP.

*C;O
"Q3

E)

*0)

o4

0

0)

0)

*s

C,;
*COb

INJECTION SITE TUMOURS AND PRECEDING PATHOLOGICAL CHANGES

FIG. 1. Fibroblasts (f) and macrophages (m) containing ingested colouring amongst collagen fibres

at the site of 20 ipjections of Light Green SF. H & E x 300.

collagen bands, few capillaries and a
variable number of fibroblasts and macro-
phages, the latter generally containing
ingested colouring (Fig. 1). Areas of
fibroblastic proliferation were often present
in the thick collagen (Fig. 2).

The character of the lesion remained
essentially the same up to the 60th-70th
injection, with few alterations. However,
the amount of collagen progressively
increased so that on palpation the injec-
tion site was considerably thickened.
Fewer capillary structures were found in
these later lesions so that the scar tissue
presented an avascular appearance. The
population of fibroblasts was sparse and
consisted mainly of small spindle cells
(Fig. 3). In a proportion of the sites
examined there occurred foci of fibroblasts
with large conspicuous nuclei and abun-
dant cytoplasm. The morphological char-
acteristics of these fibroblasts corres-

ponded to the description in the literature
(Chapman, 1962; Ross, 1968) of young
proliferating and synthesizing fibroblasts.
Ultrastructural examination revealed that
such cells had abundant, extensive, rough
endoplasmic reticulum, with large groups
of attached ribosomes, randomly located
Golgi and large mitochondria with exten-
sive cristae. The nuclei were large with
prominent but structurally normal nucleoli
(Fig. 4). Frequent mitotic figures were
observed in these foci, indicative of
proliferative activity.

Other features less commonly encoun-
tered were the formation of cysts con-
taining an eosinophilic proteinaceous
exudate and foci of inflammatory cell in-
filtration, chiefly lymphocytic in character.

Malignant tumours were seen micro-
scopically after 65-70 injections in a
proportion of the animals. These were
small lesions and consisted of an interlac-

23 3

FIG. 2.-Foci of proliferating fibroblasts (f) in heavily collagenized subcutaneous tissue, given 53

injections of Blue VRS. H & E x 300.

FIG. 3.-Quiescent heavy collagenization (c) of the subpannicular connective tissue layer at the

site of 60 injections of Blue VRS. H & E x 120.

FIG. 4.-Electron micrograph of fibroblast from site of 40 injections of Blue VRS in rats, showing

morphologically normal nucleus and nucleolus. Pb citrate/uranyl acetate x 30,000.

FiG. 5.- Morphologically abnormal fibroblasts, indicative of early sarcomatous change, at the site

of 65 injections of Light Green SF. H & E x 120.

FIG. 6.-Injection site sarcoma in a rat given 90 injections of Blue VRS. H & E x 720.

FIG. 7. Resolution of scar tissue in rat given 20 injections of Light Green SF and allowed to survive

until the termination of the experiment. H & E x 120.

. .  . .    ........... .                                  .          ..      .      .  ..

..... .... ...

....                                    . .                   .. ..

.... ...... .

......... .. M.

. .....                  . .          ....

INJECTION SITE TUMOURS AND PRECEDING PATHOLOGICAL CHANGES

ing network of spindle cells, amongst
which morphologically abnormal cells
were present (Fig. 5). This histological
picture conformed to the early sarco-
mata described by Carter (1969). In
some instances there was evidence of
invasion of adjacent muscle, establishing
beyond doubt the malignant nature of
these tumours from an early stage.

In rats that had received more than
70 injections the histology of all sites
examined could be fitted into one of 3
categories. The first consisted of those
lesions which were composed of extensive
collagenization and foci of fibroblastic
proliferation. The second category of
lesions exhibited areas of early sarco-
matous change microscopically and in the
third category frank sarcomata were
present (Fig. 6) macroscopically forming
nodular masses 2-4 cm in diameter.

(b) Rat.s injected for only part of the
experimental period. Animals given up to
25 injections of Light Green SF or Blue
VRS and then allowed to survive until
the termination of the experiment showed
no macroscopical abnormality at the
injection site. Histologically, no differ-
ence could be detected between these
samples and a control uninjected site.
Evidently the scar tissue produced in
response to the early injections had
resolved (Fig. 7).

No resolution was observed in any rat
that had received more than 25 injections,
although areas of fatty tissue could be
seen between the collagen strands in
animals receiving less than 40 injections.

In rats that had received more than
40 injections thick scar tissue containing
wide collagen bands filled the subcu-
taneous area, and foci of fibroblastic
activity, similar to those described in
the earlier section, were seen in several
animals. Other animals had developed
sarcomata locally. At all stages of the
experiment, a heavy macrophage response
was evident after injections of Light
Green SF. This response was less con-
spicuous in the lesions induced by Blue
VRS.

16

The principal pathological findings in
these experiments are summarized in
Table II.

Tissue reaction to carcinogens

(a) Rats injected throughout the experi-
mental period.-The initial responses of
the subcutaneous tissue to the injection
of the carcinogenic agents MNU and NQO
have been reported earlier (Hooson and
Grasso, 1971). The lesions seen in rats
given up to 24 injections did not differ
markedly from these. Extensive destruc-
tion of the subcutaneous tissue was
evident and the site was filled mainly
with a haemorrhagic exudate in which
fibrinoid fibrillar material containing occa-
sional histiocytes and polymorphs were
present (Fig. 8). Typical granulation
tissue was absent but around the area of
destruction some fibroblastic prolifera-
tion was taking place; a few capillary
sprouts were in evidence. The fibroblasts
showed extensive variation in size and
shape and an abnormal nuclear morpho-
logy was often observed (Fig. 9).

An early sarcoma was seen histologic-
ally in a rat that had received 40 injec-
tions. After the 45th injection the
majority of the injection sites were found
to contain tumours of either the connective
tissue or the mammary gland tissue (Fig.
10). In the remaining few animals,
the histology did not differ from that
described above. Mammary ducts showed
some degree of hyperplastic activity,
exhibited by increased mitotic activity,
and the formation of several layers of
epithelial cells at irregular intervals along
the lining of the dilated ducts from the
24th injection onwards.

(b) Rats injected for only part of the
experimental period.-Only 12 of the 55
rats in this group did not bear neoplasms
at the injection site. Animals in this
series conspicuously developed tumours
after very short exposure to carcinogens.
For example, adenocarcinomata and sarco-
mata were recorded 35-41 weeks after
the injection of only 4 doses of NQO. No
local thickening was observed at the

237

J. HOOSON, P. GRASSO AND S. D. GANGOLLI

--O O00 O e 'O  Xt OeO OOC o

000001000 00000_000
---------- \?>1 - - -- --  --mK_

e   co   o t- (   0   eq I-
t4 iC t'       _ c- eq  Co =  -

eq            -4             P-

C0 l 00             cOo

P XO            e

O   4  C  eq  e  4   e  Co   C o

eq  t   r'  os   -  e q  10 -  0

4 C O 4 I e 1 0

N"W t o O cse

4-D       4Q         4-D Q

o          C         0         C)

0          0       -N          0

V-1       IL-4     W 014       3

-Q-Q 1        %  -14

0
0
4a

0
aD

0 0
o 4

4-Q

Ca

0

0
*4   0

"g" *-

S

0    Ca
O    -Q
00

Caa

(  4_

*4-4

0

4a
_a   0

0 0 " C
o oO
*?

0
W z
,  _ *

.a OCa>

Oc

( 3+

m

4a
0
C)
k
C)
0
0)

t-Q

4-Z

-Q

~0
0
114

0
0

0

00
*_ m

C) 0
C))

O .

C)

*: t

238

V

0s
GO

CO

GO2t

V *S

.~ V

GO

V
GO

0t
GO
Vt

Vt
GO

0 S
V

S
V

14.5.

Ho

k.5

7a ~

E? (a

0m

D

f 4 *-

Z._)

FIG. 8.-Absence of granulation tissue formation or reparative processes operative in the subcutaneous

site after 20 injections of MNU in rats. H & E x 120.

FiG. 9.-Electron micrograph of fibroblast from site of 20 injections of NQO in rats. The nucleus

is dense and segregation of nucleolar elements is obvious.. Pb citrate/uranyl acetate x 25,000.

J. HOOSON, P. GRASSO AND S. D. GANGOLLI

FIG. 10.-Cystic mammary adenocarcinoma at the site of 40 injections of methylnitrosourea in a rat.

H&E x120.

injection site unless sarcomata were
present. Histologically, the injection
site contained a thin layer of connective
tissue with normal-looking fibroblasts
and a slight cellular infiltrate of mono-
nuclears and histiocytes.

The principal pathological findings in
these experiments are summarized in
Table III.

Induction of tumours

In Experiments 4 and 6, designed
specifically to assess the tumorigenic
response, 19 out of 20 rats treated with
MNU developed local tumours (11 sarco-
mata and 8 mammary adenocarcinomata).
The adenocarcinomata occurred predo-
minantly in females, SPF/CSE females
being particularly susceptible.

Of the 16 rats which survived to
tumour bearing age after injection of
NQO, 10 developed local sarcomata and
4 carried mammary tumours (Table I).

Morphology of injection site tumours

The sarcomata consisted histologically
of interlacing bundles of spindle cells.
The bundles varied in thickness from a few
to several cells. In the differentiated
tumours the bundles were clearly defined
and were made up of cells closely resemb-
ling young immature fibroblasts. Intra-
cellular matrix was abundant and consisted
of strands of reticulin and collagen. The
resemblance of cells and fibres to connec-
tive tissue became more indistinct with the
progressive loss of differentiation until the
tumour cells lost completely their resemb-
lance to the cell of origin and displayed
considerable pleomorphism. Despite this
range of histological appearance, all the
tumours were locally infiltrative, destroy-
ing surrounding muscle and overlying
skin. No metastases were found.

The mammary adenocarcinomata were
either solid or papillary. The solid type
consisted of areas packed with small

240

INJECTION SITE TUMOURS AND PRECEDING PATHOLOGICAL CHANGES  241

1Q

*S rt  |  ~o               0 't ??

*t~~~~~~ 't                                 E >O*  - q00* >C

t s_tCO  4_                                   0 C

0             X                         <9

~~ Po       o     oo     o      xXo~~~~~~

7~~~~~~~~~~~~~~~~~~~~-

W    <            fx~~~~~~~~~~~~~~~~~~~~~~~C

?z

EZQI.                    C O   OCO

J. HOOSON, P. GRASSO AND S. D. GANGOLLI

acini or covered with sheets of epithelial
cells. The cells were small with a promi-
nent nucleus and displayed a variable
amount of pleomorphism and mitotic
activity. Again, no metastases were
found in this experiment.

DISCUSSION

The results presented in this paper
demonstrate that the difference in histo-
logical response between the injections of
surfactants and carcinogens extends from
the first few weeks through to the develop-
mnent of tumours. The resultant neoplas-
tic responses are also in strong contrast
to each other, as described later.

Tissue reaction to surfactant colourings

The essential histological findings of
progressive collagenization and continued
proliferative fibroblastic response in the
above experiments with surfactants have
also been described at the site of repeated
injection of hypertonic glucose (Takizawa,
1940; Cappellato, 1942) in the connective
tissue envelope that develops around
plastic films (Oppenheimer et al., 1959)
and after administration of Fe dextran
(Baker et al., 1961) or NTDQ, a rubber
additive (Carter, 1969).

In these cases, the incidence of sarco-
mata recorded is comparable with the
high incidence of sarcomata observed in
our experiments with the surfactant
colourings. Progressive fibroblastic pro-
liferation seems to be a vital factor in the
development of these sarcomata.

Carter, Birbeck and Roberts (1970)
stated that enhanced premitotic activity,
measured by incorporation of [313] thymi-
dine, continued for 40 weeks after multiple
injections of NTDQ. Previous work in
our laboratories has shown that colourings
devoid of those physical factors known to
induce massive local necrosis produce,
when injected subcutaneously, a self-
limiting lesion in which the connective
tissue acquires histological features of
maturity. In long-term tests these did

not give rise to local sarcomata (Grasso
and Golberg, 1966b).

It is not easy to explain why a conti-
nuous proliferative activity of fibroblasts
leads to malignancy. A high cellular
turnover is associated with an increase in
mutation rate, and therefore with a subse-
quent higher risk of the emergence of
malignant cells (Atwood and Scheinberg,
1958). It is possible that some mutant
cells have initially some selective advan-
tage over normal fibroblasts, enabling
them to survive rather than be eliminated
as would be most mutants (Vasiliev et al.,
1962). However, other factors also can
play a part in the development of neo-
plasia. Several   investigations  have
stressed the importance of a sustained
derangement of the microenvironment in
favouring the development of local sarco-
mata. It is feasible that the thick colla-
gen which develops at the site of the
injection could isolate fibroblasts from
the regulators of cell growth, such as
cell-cell contact, biochemical exchanges
and perhaps immunological processes
(Carter, 1969).

A similar formation of hyalin collagen
is seen when implants of solid materials
are made into the subcutaneous tissue
of rats, with sarcomata as the ultimate
outcome. Histological descriptions of the
connective tissue reaction around silicone
rubber implants (Nothdurft, 1961) and
various plastic films (Oppenheimer et al.,
1959) emphasize the importance of the
formation of a thick, avascular connective
tissue capsule, with consequent isolation
of the fibroblasts, as an essential step in
the evolution of malignancy.

It is significant that in the injection
experiments reported in the present paper,
no tumours arise where the local tissue
returns to its normal architecture, for
example in rats taken off treatment after
up to 24 injections of surfactant. The
same phenomenon was observed in sequen-
tial studies of the connective tissue lesion
that develops around plastic films. If
the film was removed surgically after 10
weeks of implantation, complete absorp-

242

INJECTION SITE TUMOURS AND PRECEDING PATHOLOGICAL CHANGES

tion of the connective tissue capsule
occurs and no sarcomata arise (Druckrey,
1960). If surgical removal is delayed
until 20 weeks, the scar tissue persists
and sarcomata eventually develop (Oppen-
heimer et al., 1958).

Reactions to carcinogens

A great contrast to these observations
was seen when carcinogens were injected
into rats. No proliferative lesions occurred
but a totally different response was
immediately apparent. In these cases, the
intermediate stage of the tissue reaction
was an extension of the initial reaction
elicited during the first 5 weeks of treat-
ment, and thus the predominant feature
was an inhibition of fibroblastic prolifer-
ation rather than a stimulation to divide.
This damping effect persisted histologically
to a stage immediately before the appear-
ance of neoplasia at the site.

Similar findings have been recorded
previously by Vasiliev et al. (1962) when
implanting paraffin pellets containing
DMBA subcutaneously. Connective tissue
proliferation was inhibited and fibroblastic
differentiation  was  depressed. Other
carcinogenic polycyclic hydrocarbons have
been reported to inhibit the connective
tissue response when injected subcu-
taneously in oil (Orr, 1939; Shabad,
1935; Wolbach, 1936). In the action of
carcinogens, a different mechanism respon-
sible for malignant change seems to be in
operation and a reaction between an
active form of the chemical and a cellular
receptor site responsible for cell growth
has been postulated.

Comparison of neoplastic response

1. Latent period.-Several other fac-
tors deserve consideration when comparing
the different responses elicited by carcino-
gens and surfactant colourings. One of
these is the induction period for tumori-
genesis. When the colourings were used,
80-90 twice weekly injections were the
usual number administered before neo-
plasia occurred. Other workers have

reported similar time intervals with other
compounds producing malignant change
by a process of repeated fibroblastic
proliferation. Thus, local tumours induced
by daily injection of hypertonic glucose
in rats and mice appeared by 45-50 weeks
(Capellato, 1942) and sarcomata induced
by sorbic acid were characterized by a
long induction period of 52-82 weeks
(Dickens, Jones and Waynforth, 1966,
1968). In contrast, injections of MNU
and NQO elicited local sarcomata as early
as 19 weeks from the beginning of treat-
ment. This reduced time interval is
in accordance with the experience of other
workers. Repeated injection of several
polycyclic aromatic hydrocarbons resulted
in local tumours by 12-20 weeks (Bonser
and Orr, 1939). The potencies of MNU
and NQO are emphasized when off-
treatment animals are taken into consi-
deration. Only 4 injections of NQO and
16 injections of MNU gave rise to eventual
malignant connective tissue tumours
(Table III). Usually around 60 injections
of the surfactant colourings were necessary
before sarcomata could be induced locally
(Table II).

Dose of chemical

The dose of chemical required to pro-
duce a carcinogenic effect is another
important distinguishing feature of the
types of response under discussion. At
the termination of the surfactant experi-
ments 1-1 5 g of dye had been adminis-
tered to rats. With the carcinogens the
dose was much smaller. About 40 mg
of MNU and 20 mg of NQO were given to
rats. Thus the difference in dosage neces-
sary to induce neoplasia is considerable.

Mammary tumour production

A completely new tumorigenic response
was elicited by MNU and NQO which has
no parallel in the experiments with Light
Green SF and Blue VRS. A high percen-
tage of mammary tumours were produced
locally, the majority in female rats.
Again, a short latent period of 12-13 weeks

243

244             J. HOOSON, P. GRASSO AND S. D. GANGOLLI

characterized the formation of these
tumours and very small doses of both
carcinogens (4-6 injections) were shown
to induce them.

CONCLUSION

It appears that there are certain clear
cut differences in the quality of the
neoplastic response which, when taken
in conjunction with the early and inter-
mediate histological changes at the site of
injection, render it possible to distinguish
between sarcoma production denoting a
true carcinogenic response and sarcomata
arising from a derangement of connective
tissue repair.

REFERENCES

ATWOOD, K. C. & SCHEINBERG, S. L. (1958) Somatic

Variations in Human Erythrocyte Antigens. J.
cell. comp. Phy8iol., Suppl. 1, 97.

BAKER, S. B. DE C., GOLBERG, L., MARTIN, L. E. &

SMITH, J. F. (1961) Tissue Changes Following
Injection of Iron-Dextran Complex. J. Path.
Bact., 82, 453.

BONSER, G. M. & ORR, J. W. (1939) The Morphology

of 160 Tumours Induced by Carcinogenic Hydro-
carbons in the Subcutaneous Tissues of Mice. J.
Path. Bact., 49, 171.

CAPELLATO, M. (1942) Sui sarcomi sperimentali da

glucosio nel ratto bianco. Tumori, 16, 38.

CARTER, R. L. (1969) Early Development of Injec-

tion Site Sarcomas in Rats; a Study of Tumours
Induced by a Rubber Additive. Br. J. Cancer,
23, 408.

CARTER, R. L., BIRBECK, M. S. C. & ROBERTS, J. D.

B. (1970) Development of Injection Site Sarco-
mata in Rats; a Study of the Early Reactive
Changes Evoked by a Carcinogenic Nitrosoqui-
noline Compound. Br. J. Cancer, 24, 300.

CHAPMAN, J. A. (1962) Fibroblasts and Collagen.

Br. med. Bull., 18, 233.

DICKENS, F., JoNEs, H. E. H. & WAYNFORTH, H. B.

(1966) Oral, Subcutaneous and Intratracheal
Administration of Carcinogenic Lactones and
Related Substances: the Intratracheal Adminis-
tration of Cigarette Tar in the Rat. Br. J.
Cancer, 30, 134.

DICKENS, F., JONES, H. E. H. & WAYNFORTH, H. B.

(1968) Further Tests on the Carcinogenicity of
Sorbic Acid in the Rat. Br. J. Cancer, 22, 76.

DRUCKREY, H. (1960) Berliner Symposium uber

Fragen der Carcinogenese. Abh. dt. Akad. Wis8,
Berl., Klasse Med., 3, 98.

GRASSO, P., GANGOLLI, S. D., GOLBERG, L. &

HoosoN, J. (1971) Physico-chemical and Other
Factors Determining Local Sarcoma Production
by Food Additives. Fd. Cosmet. Toxicol., 9, 463.
GRASSO, P. & GOLBERG, L. (1966a) Early Changes at

the Site of Repeated Injection of Food Colourings.
Fd. Cosmet. Toxicol., 4, 269.

GRASSO, P. & GOLBERG, L. (1966b) Subcutaneous

Sarcoma as an Index of Carcinogenic Potency.
Fd. Cosmet. Toxicol., 4, 297.

HoosoN, J. & GRASSO, P. (1971) Early Reactions of

the Subcutaneous Tissue to Repeated Injections
of Carcinogens in Aqueous Solutions. Br. J.
Cancer, 25, 505.

NOTHDURFT, H. (1961) Sarkomergengung bei

Ratten durch implantierte Fremndkorper. Ther.
mh., 8, 262.

ORR, J. W. (1939) An Investigation of the Histo-

logical Changes of the Subcutaneous Tissues of
Mice during the Induction of Sarcoma by Carcino-
genic Hydrocarbons. J. Path. Bact., 49, 157.

OPPENHEIMER, B. S., OPPENHEIMER, E. T., STOUT,

A. P., WILLHITE, M. & DANISHEFSKY, I. (1958)
The Latent Period in Carcinogenesis by Plastics
in Rats and its Relation to the Precancerous
Stage. Cancer, Philad., 11, 204.

OPPENHEIMER, B. S., OPPENHEIMER, E. T., STOUT,

A. P., DANISHEFSKY, I. & WILLHITE, M. (1959)
Studies on the Mechanism of Carcinogenesis by
Plastic Films. Acta Un. int. Canc., 15, 659.

Ross, R. (1968) The Fibroblast and Wound Repair.

Biol. Rev., 43, 51.

SCHABAD, L. M. (1935) Uber die cancerogene

Wirkung des 1: 2: 5: 6 Dibenzanthracene. Z.
Kreb8forsch., 42, 295.

SHELTON, E., EVANS, V. J. & PARKER, G. A. (1963)

Malignant Transformation of Mouse Connective
Tissue Grown in Diffusion Chambers. J. natn.
Cancer In8t., 30, 377.

TAKIZAWA, N. (1940) Experimentelle Erzeugung

des Sarkons bei der Maus durch die Injektion von
Glucose, Fructose und Galactose. Gann., 34, 1.
VASILIEV, J. M., OLSHEVSKAJA, L. V., RAIKHLIN,

N. T. & IVANOVA, 0. J. (1962) Comparative Study
of Alterations Induced by 7,12-dimethylbenz(a)
anthracene and Polymer Films in the Subcu-
taneous Connective Tissue of Rats. J. natn.
Cancer In8t., 28, 515.

WOLBACH, S. B. (1936) The Latent Period in

Experimental Carcinogenesis. Arch8 Path., 22,
279.

				


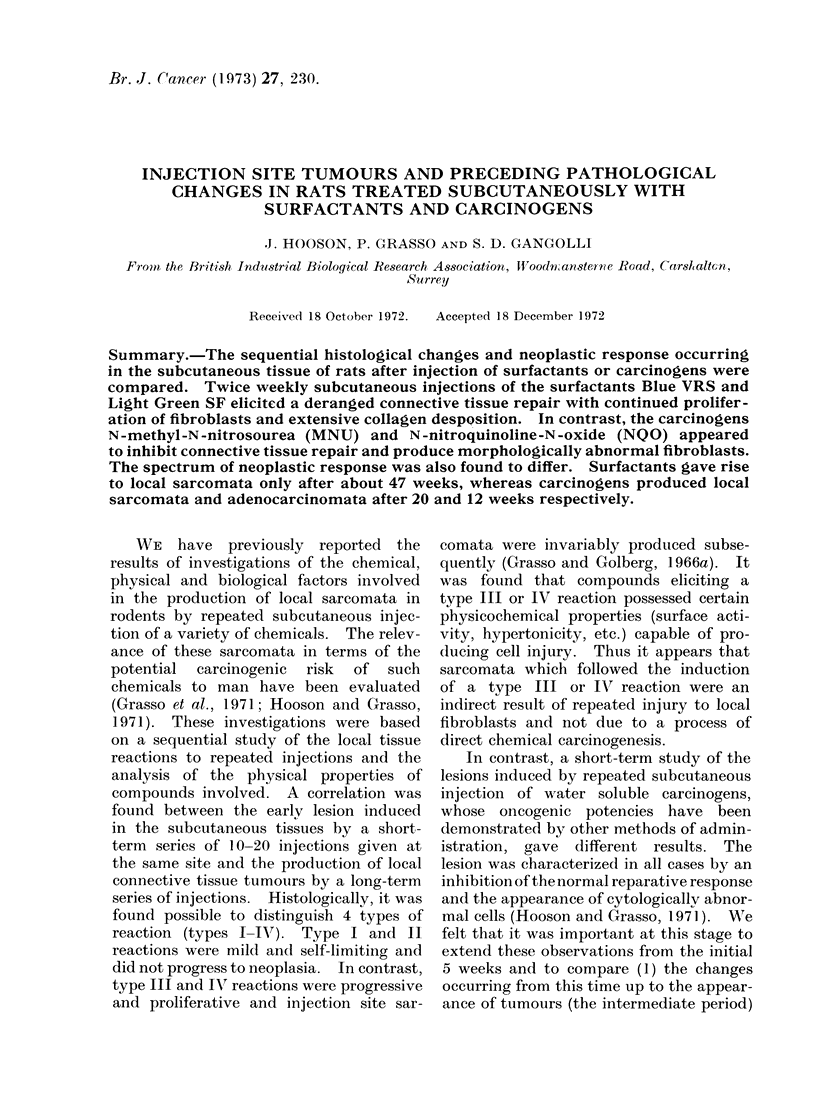

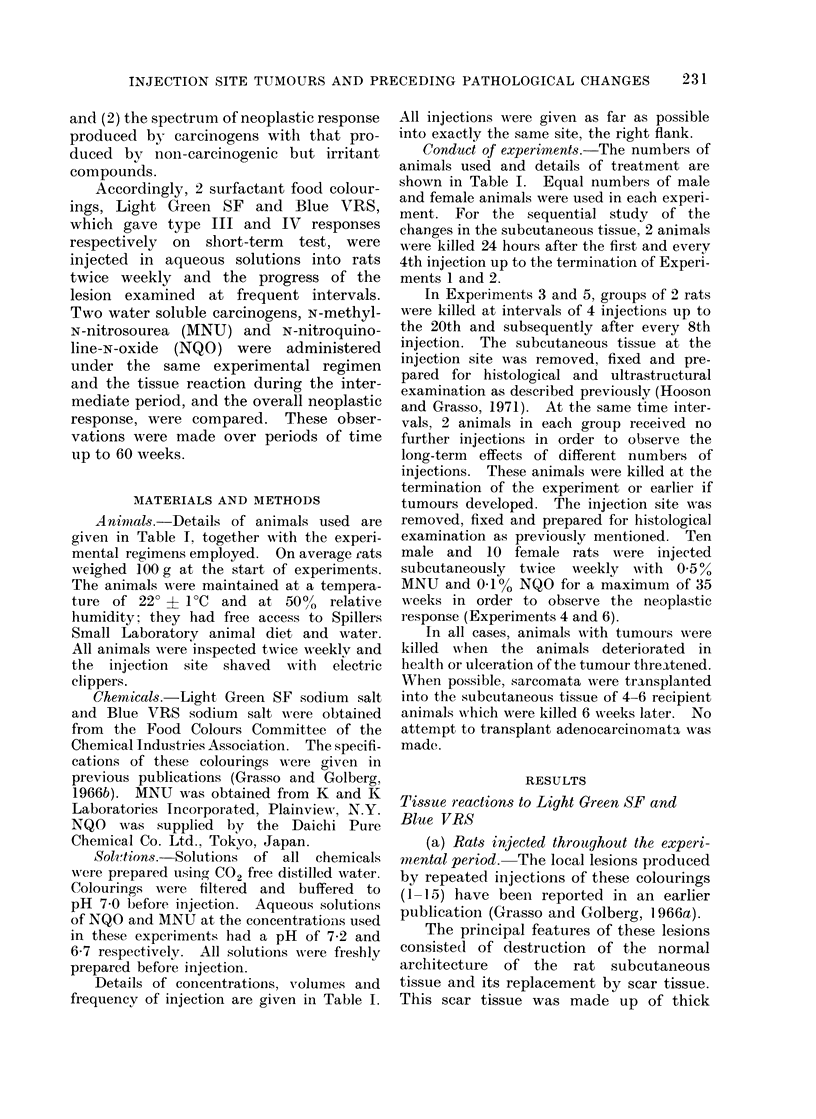

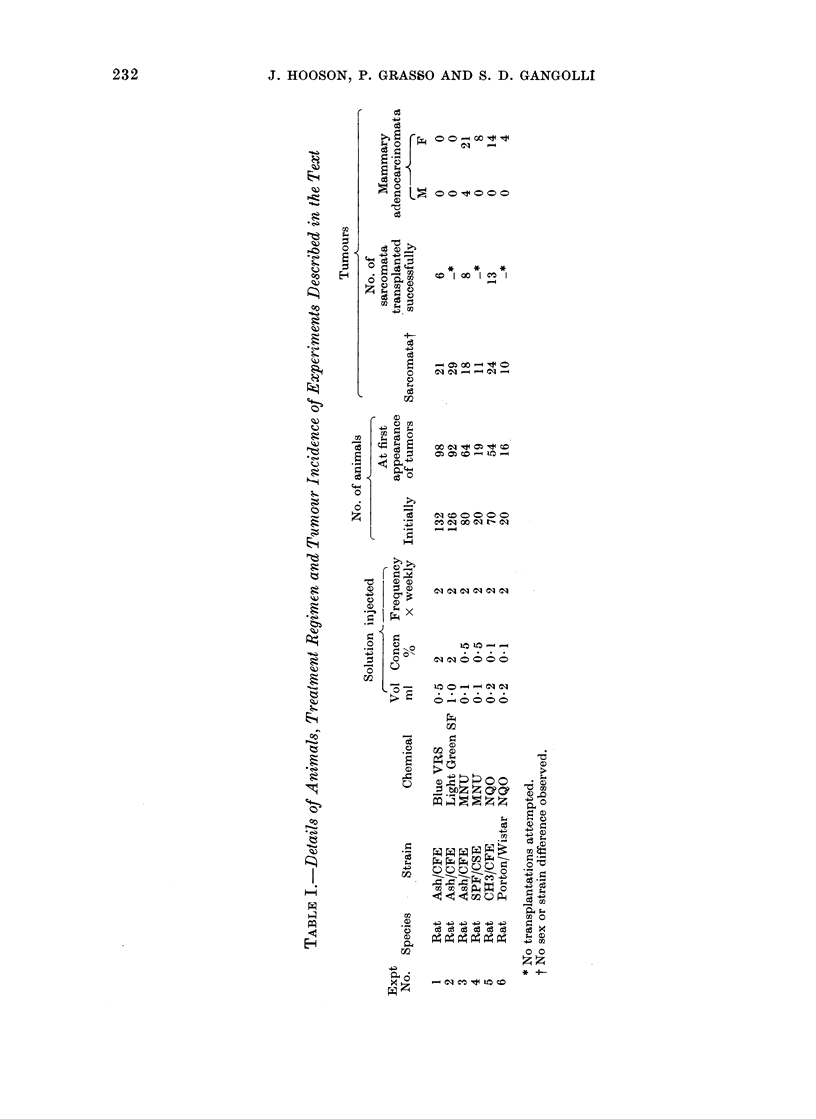

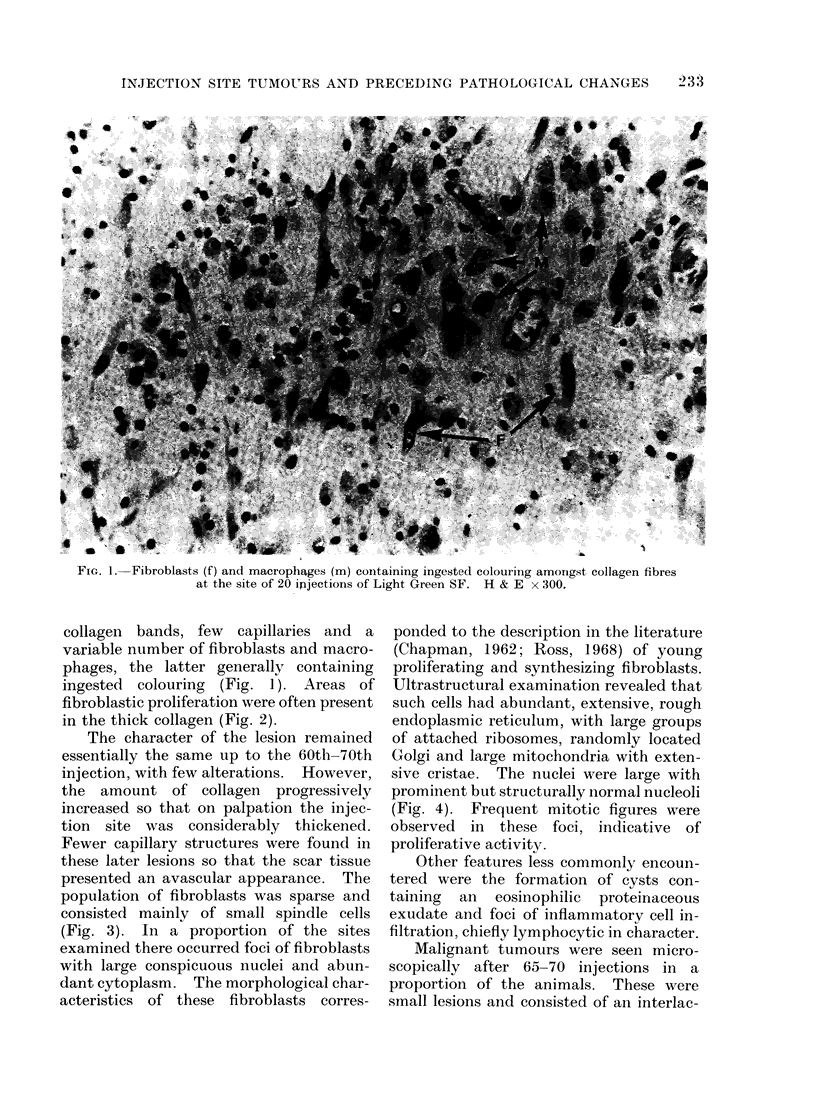

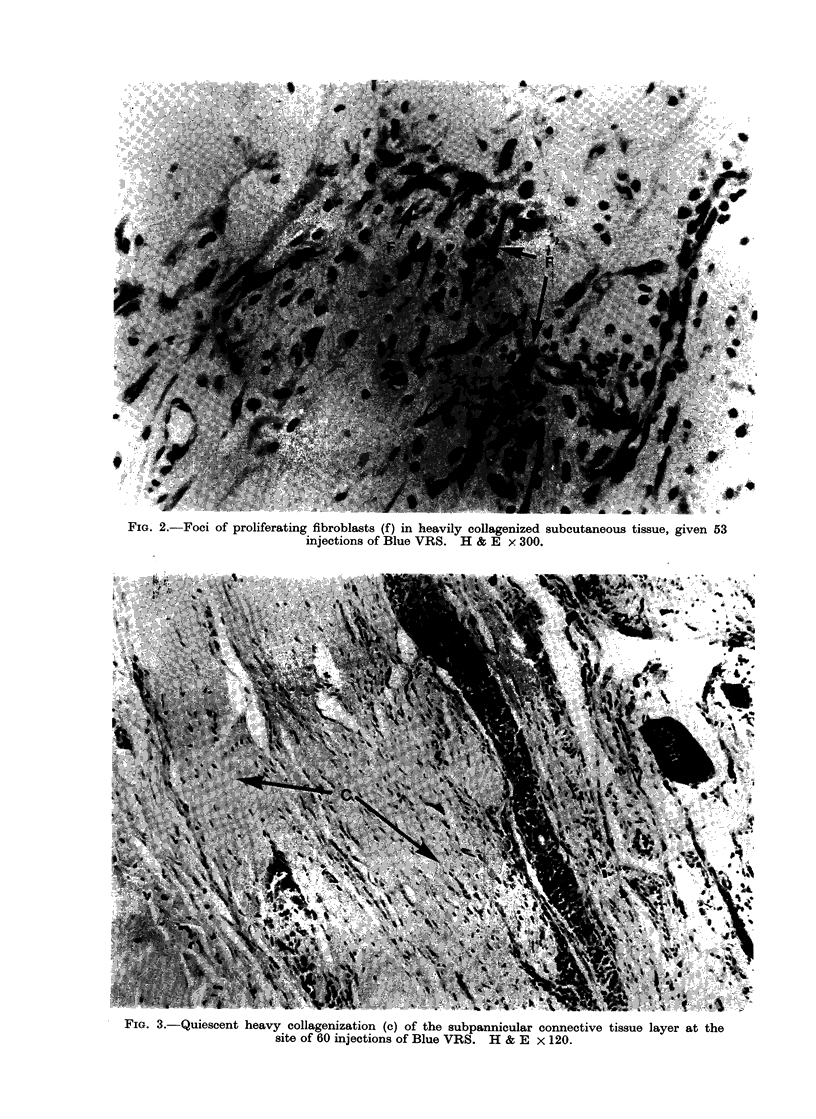

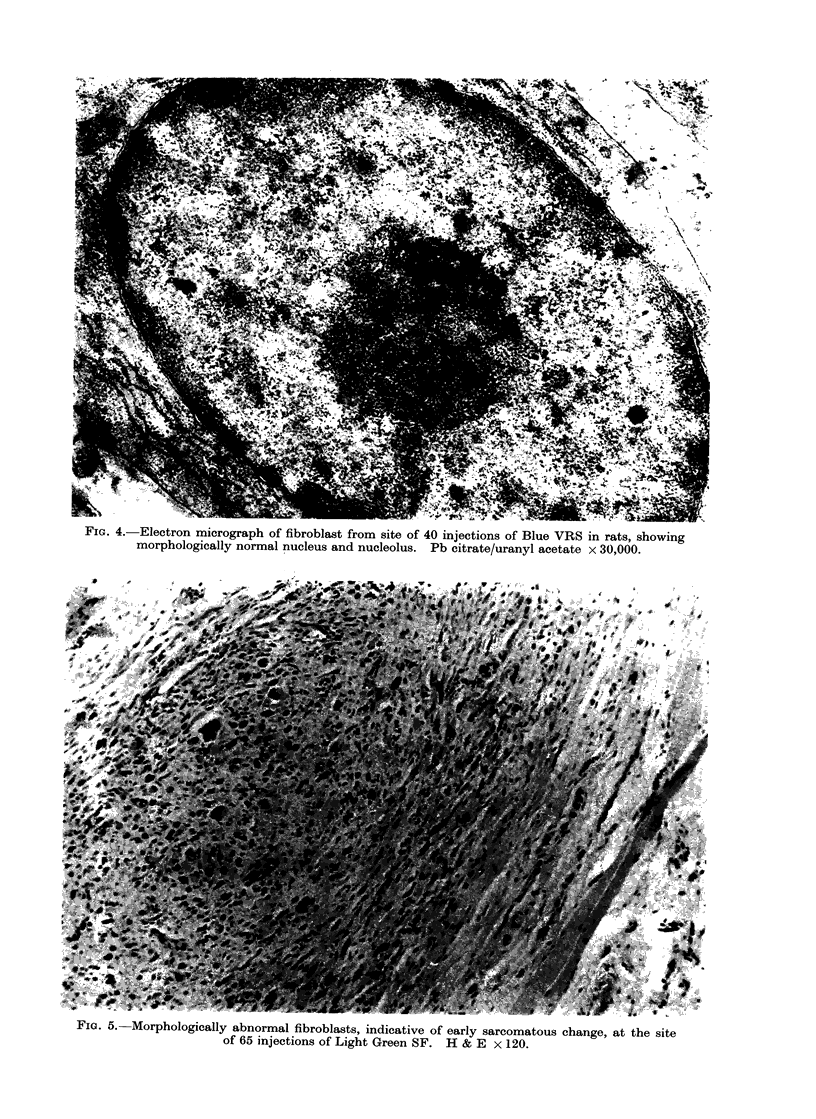

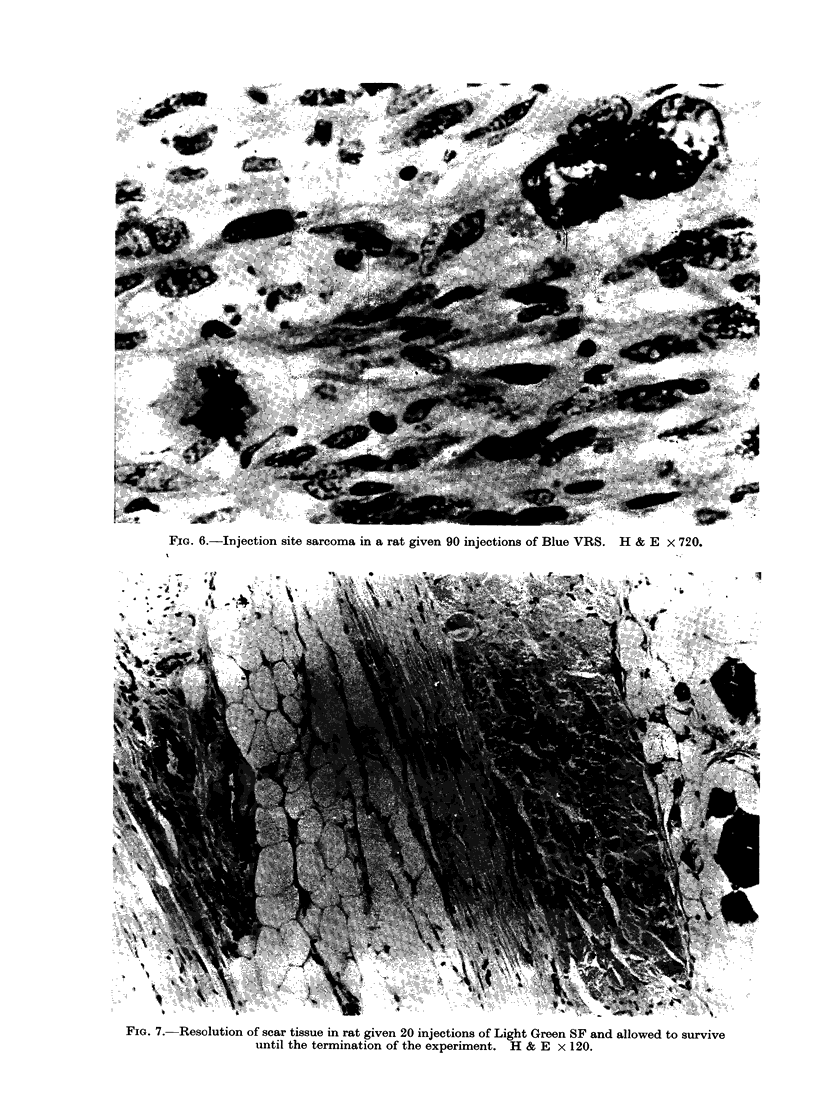

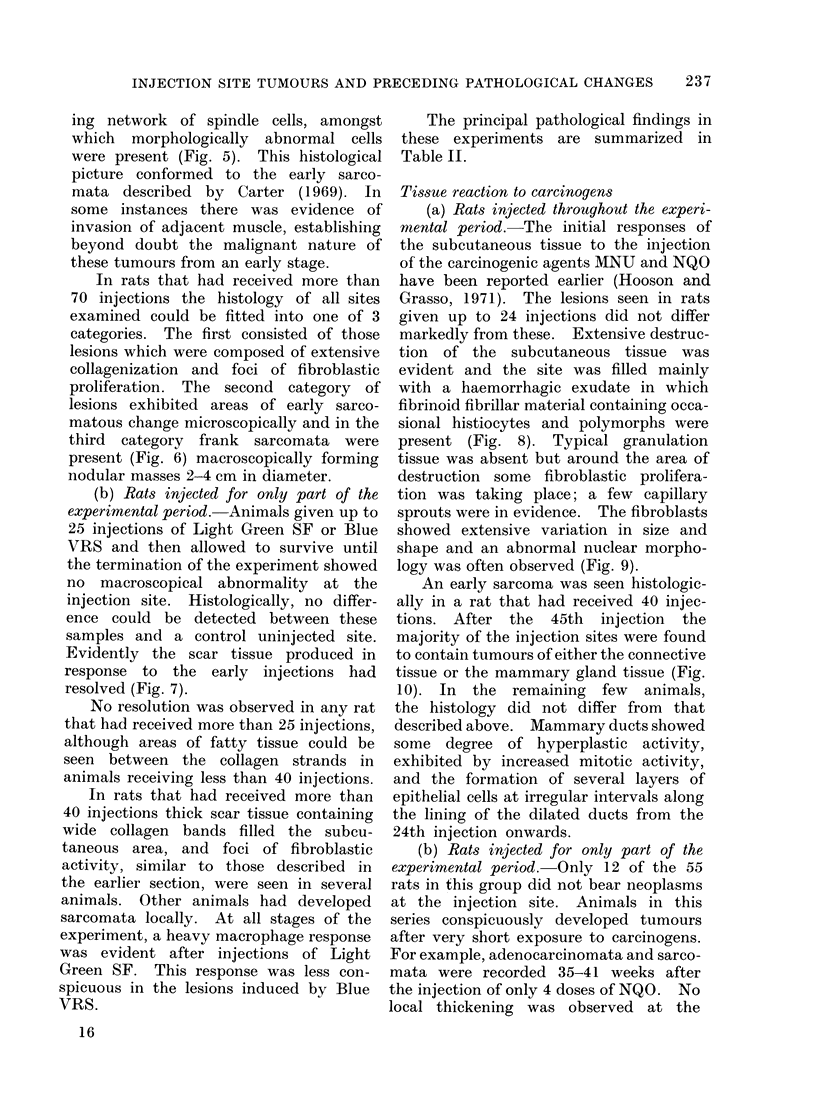

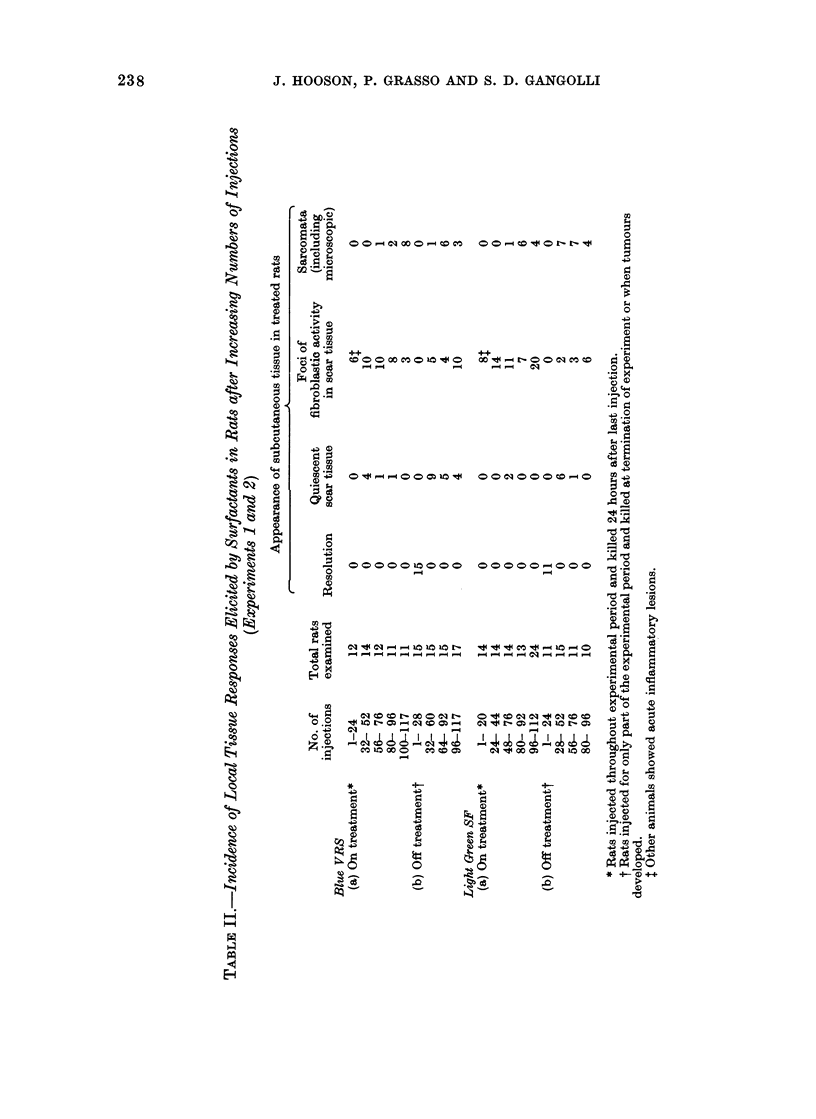

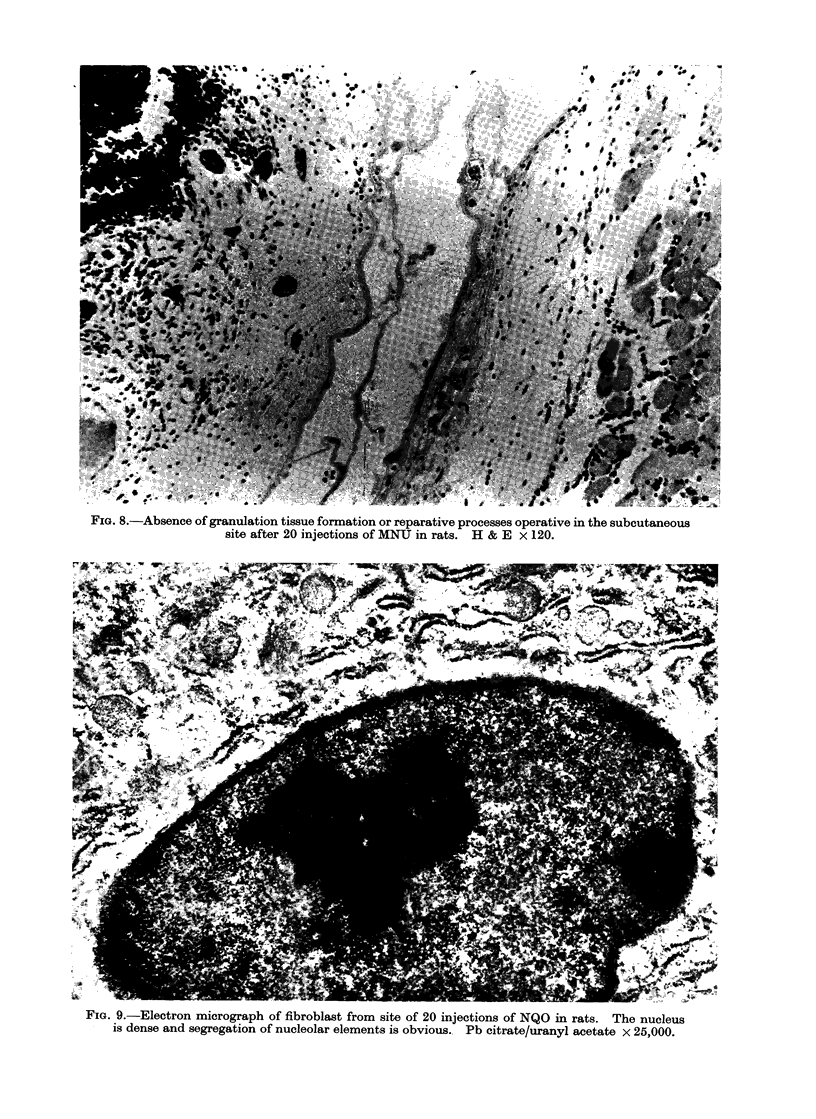

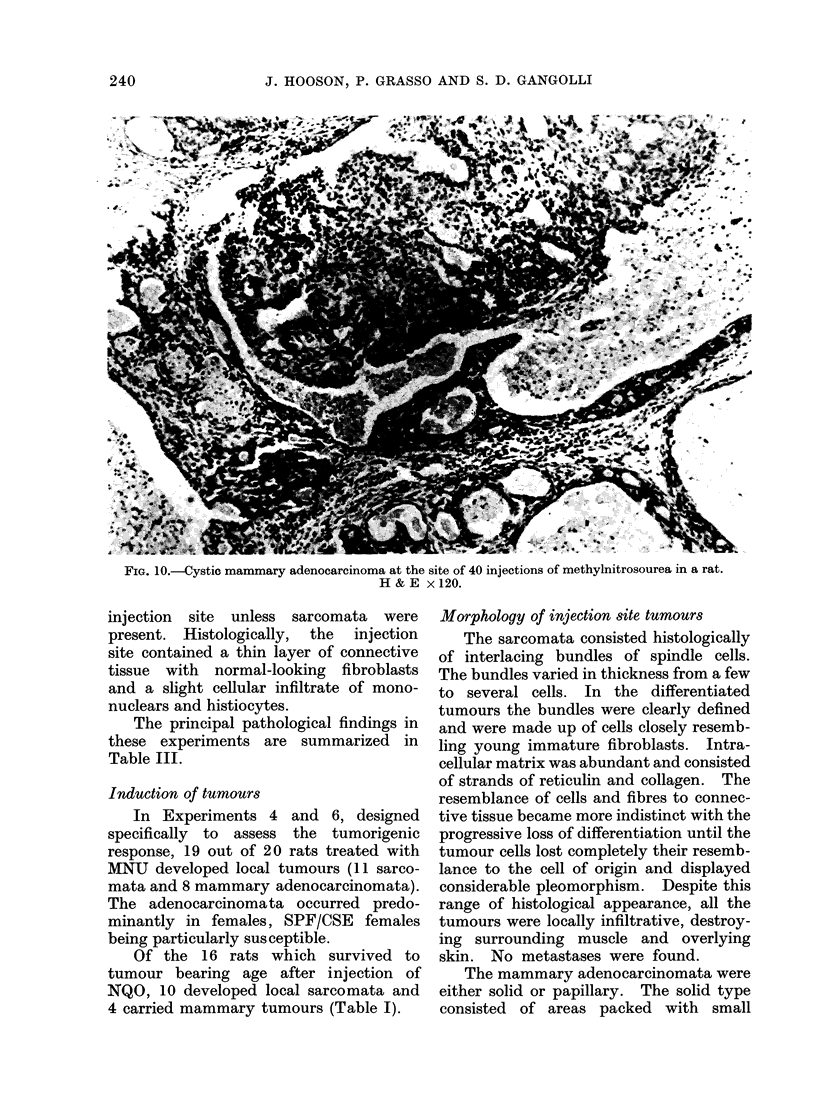

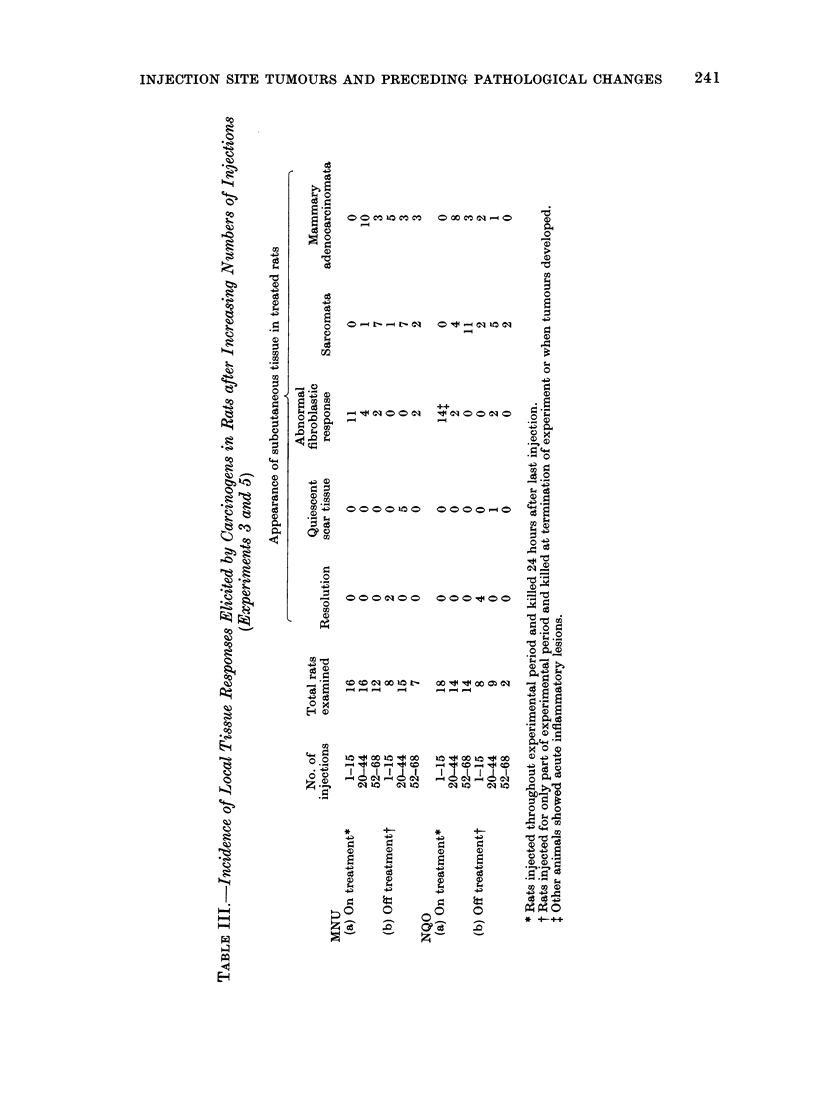

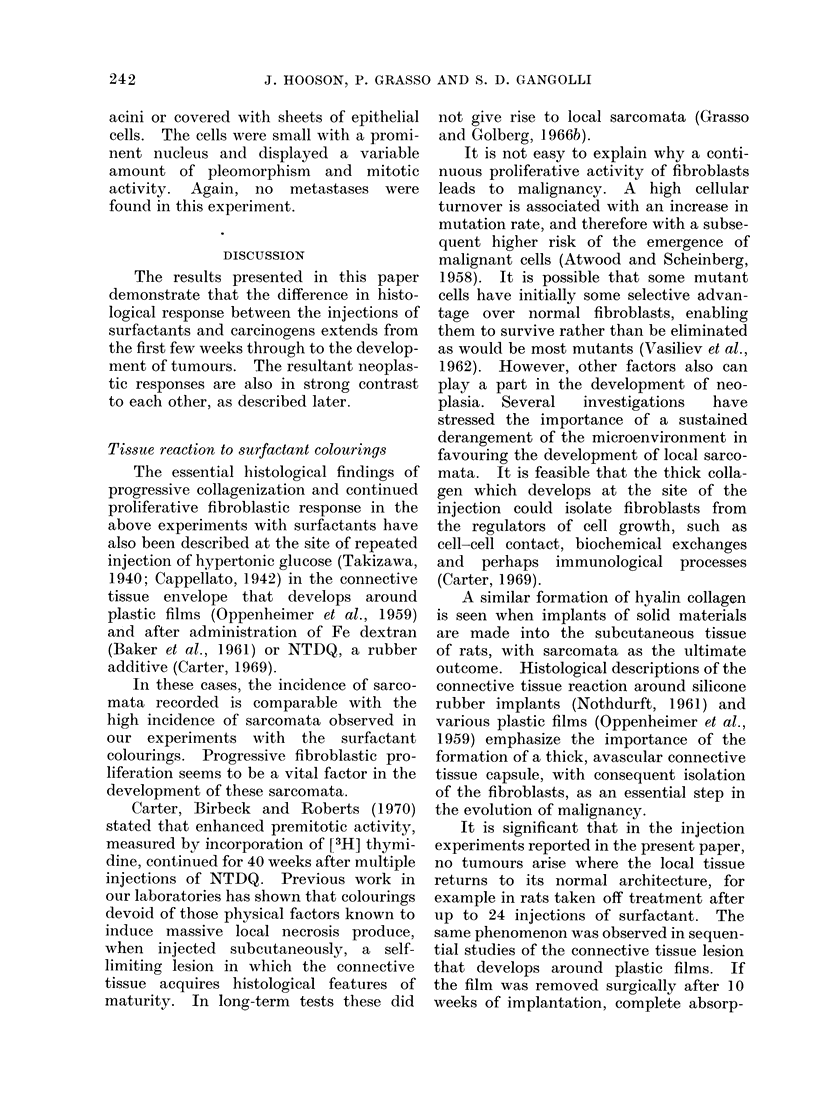

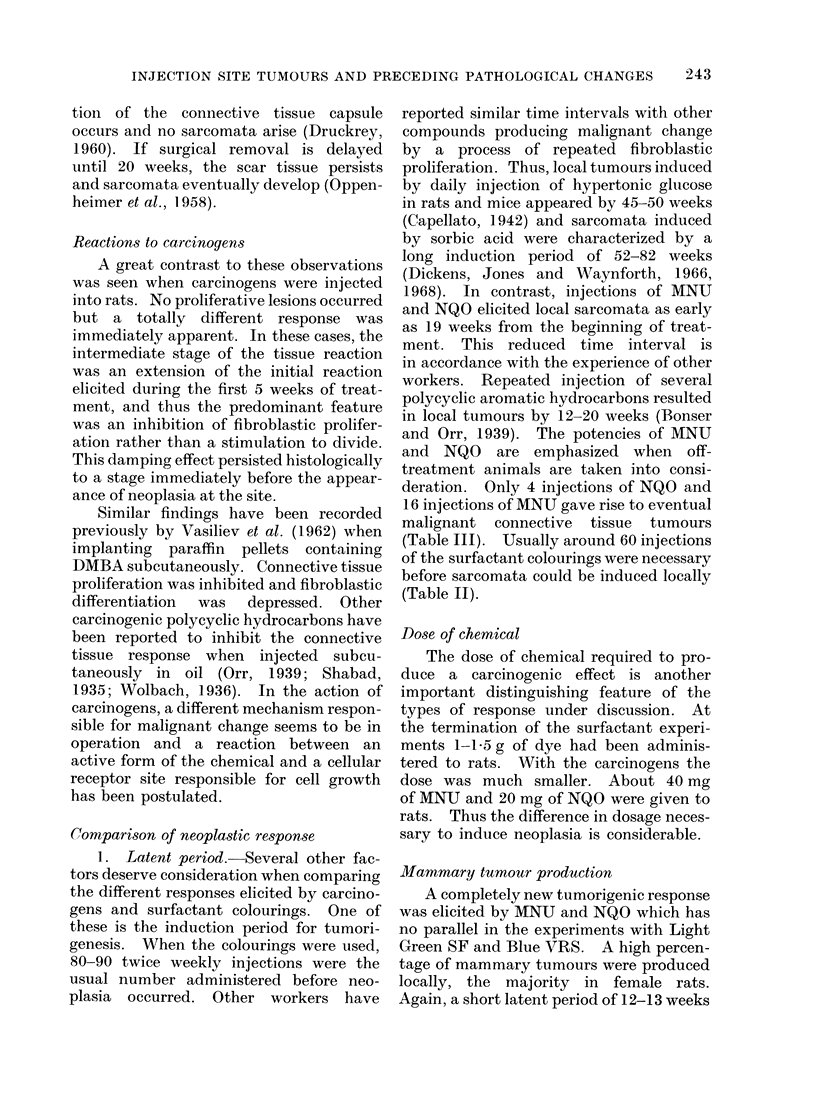

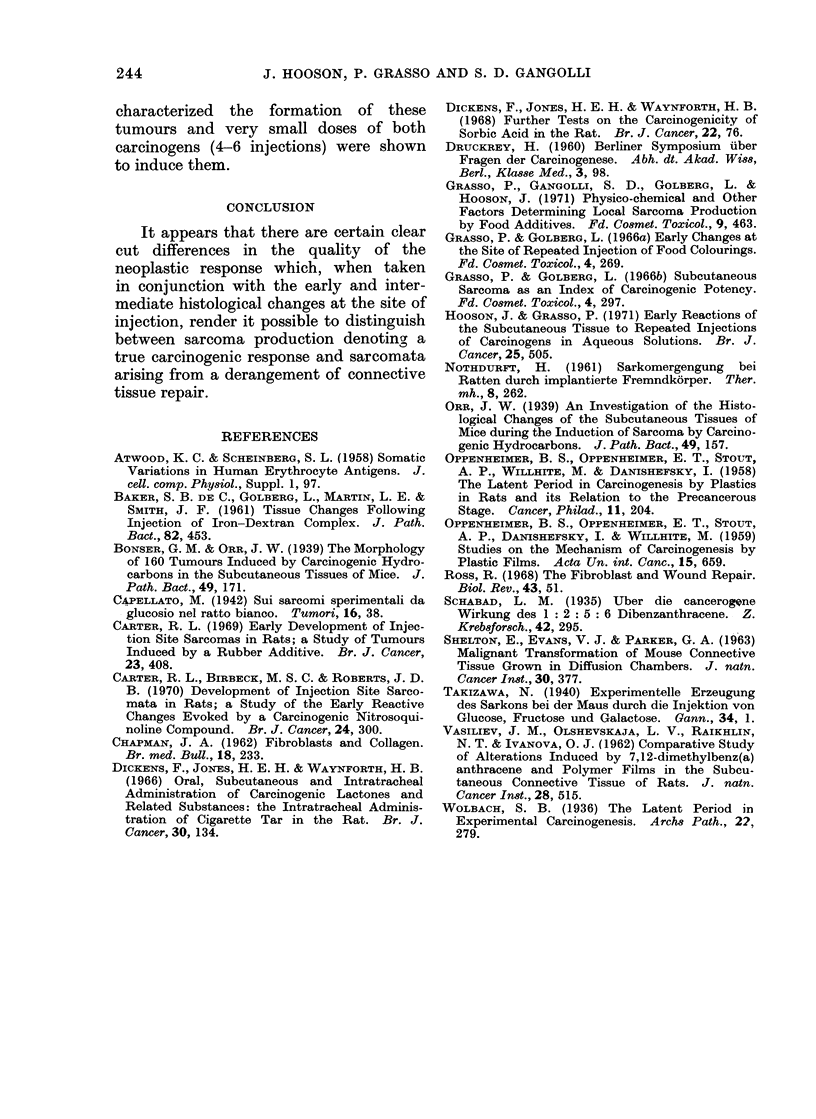

